# Trajectories of psychological and social well-being preceding death

**DOI:** 10.1136/bmjment-2025-301967

**Published:** 2025-11-07

**Authors:** Jiao Wang, Jie Guo, Abigail Dove, Xinjie Zhang, Jirong Yue, David A Bennett, Weili Xu

**Affiliations:** 1 Center of Gerontology and Geriatrics, National Clinical Research Center for Geriatrics, West China Hospital of Sichuan University, Chengdu, Sichuan, China; 2 Aging Research Center, Department of Neurobiology, Care Sciences and Society, Karolinska Institutet, Stockholm, Sweden; 3 Department of Nutrition and Health, China Agricultural University, Beijing, China; 4 Department of Neurobiology, Care Sciences and Society, Karolinska Institutet, Stockholm, Sweden; 5 Department of Pediatric Neurosurgery, West China Second Hospital of Sichuan University, Chengdu, Sichuan, China; 6 Rush Alzheimer’s Disease Center, Rush University Medical Center, Chicago, Illinois, USA

**Keywords:** Psychopathology, Mental Health

## Abstract

**Background:**

Poorer psychological and social well-being has been linked to increased mortality.

**Objective:**

To delineate the trajectories of psychological and social well-being during the last two decades of life.

**Methods:**

Within the Rush Memory and Aging Project, 1971 older adults were followed up for up to 22 years. Aspects of psychological well-being (ie, depression symptoms, loneliness and purpose in life) and social well-being (ie, cognitive activity, social activity and social network) were annually measured through structured interviews. Survival status was tracked during the follow-up period. Data were analysed using Cox regression and mixed-effect models with a backward timescale.

**Findings:**

During the follow-up, 1119 (56.77%) participants died. In multiadjusted Cox regression models, higher depression symptoms and poor social activity were associated with increased mortality. Compared with survivors, decedents showed steeper declines in psychological and social well-being, leading to significant differences up to 13 years before death for purpose in life (mean difference: −0.14 (–0.26, –0.01)), 9 years for depression symptoms (0.35 (0.10, 0.60)) and social activity (−0.16 (–0.26, –0.06)), 6 years for loneliness (0.13 (0.05, 0.21)), 4 years for social network (−1.06 (–1.77, –0.36)), and 3 years for cognitive activity (−0.12 (–0.21, –0.04)). Among decedents, the terminal phase began 11 years before death for purpose in life, 10 years for cognitive activity, 9 years for social activity and depression symptoms and 6 years for loneliness.

**Conclusions and implications:**

Psychological and social well-being may begin to exhibit terminal decline approximately 6–11 years prior to death. Longitudinal surveillance of well-being should be incorporated into the context of geriatric medical care.

**WHAT IS ALREADY KNOWN ON THIS TOPIC**

- Poor psychological and social well-being is associated with death. Although evidence supports the existence of terminal decline in well-being, the detailed patterns—particularly the rate of change over time and the timing of critical turning points—across different psychological and social domains remain inadequately characterised.

**WHAT THIS STUDY ADDS**

- Psychological and social well-being—particularly elevated depressive symptoms and decreased social activity—were associated with an increased risk of mortality.
- Trajectory analyses revealed noticeable increases in depressive symptoms and loneliness beginning 9 years and 6 years prior to death, respectively.
- At the same time, declines in purpose in life, cognitive activity and social activity were observed 11 years, 10 years and 6 years before death.

**HOW THIS STUDY MIGHT AFFECT RESEARCH, PRACTICE OR POLICY**

- Public health strategies must prioritise pre-emptive well-being preservation in older adults, leveraging this extended window for community-based engagement programmes to mitigate terminal deterioration.

## Introduction

 Older adults often experience an accelerated decline in physical and cognitive functioning – termed ‘terminal decline’^1^ – in the years preceding death, attributable to the accumulation of chronic diseases and other drivers of mortality.^2^ Ageing is a complex, multidimensional process that involves not only physical changes but also social and psychological changes. The ageing process is accompanied by changes in psychological and social well-being, including symptoms of depression, reduced cognitive and social activity, social isolation and a loss of purpose in life.^3^ In contrast, it is still unclear whether (and when) older adults’ psychological and social well-being also exhibits a phase of ‘terminal decline’ at the end of life.

### Well-being as a predictor of mortality

Several population-based longitudinal studies have pointed to an increased risk of death with poor levels of psychological and social well-being—including factors such as depression symptoms,^4^ loneliness,^5^ reduced sense of purpose in life,^6^ low social activity^7 8^ and smaller social network.^9^ In our previous studies using data from the Rush Memory and Aging Project (MAP), we found that several indicators of psychological and social well-being were associated with mortality.^10^ In contrast, other studies have reported that loneliness,^11 12^ depressive symptoms^12^ and social network size^13^ were not significantly associated with mortality. These inconsistent results may be due to differences in sample characteristics and statistical methods and, more significantly, may be influenced by the length of follow-up. Additionally, several studies have identified significant sex differences in the association between well-being and mortality,^12 14^ possibly due to differences in physiological features and social relevance.^14^

### Terminal decline in well-being

Traditional cohort study designs and time-to-event analysis approaches have unavoidable limitations, including a restricted follow-up time and variation in exposures throughout follow-up. Notably, by anchoring the time scale to a long terminal phase before death (rather than just the baseline assessment), the trajectory of exposure can be more clearly reflected, allowing for capturing more information about longitudinal changes in exposure. Although previous evidence reveals that there may be terminal decline in well-being,^15^,^20^ the trajectories of decline (especially the time windows in which the decline begins) and the differences in the change patterns across different well-being indicators remain insufficiently elucidated.^16^ In addition, sex-specific patterns and long-term trends have not been well characterised.

In the present study, we used 22 years of data from the MAP, a community-based cohort study of older adults with annual follow-up, to examine the interplay between changes in psychological and social well-being and mortality by simultaneously examining a broader spectrum of well-being indicators and using a unified statistical approach aligned with the time-to-death framework. Specifically, we conducted two analyses: (1) time-to-event analyses to examine the extent to which between-person differences in psychological and social well-being are associated with mortality and (2) retrospective trajectory analyses to compare within-person changes in psychological and social well-being during the last two decades of life.

## Methods

### Study populations

MAP is an ongoing longitudinal clinical-pathologic cohort study initiated in 1997, involving older adults recruited from continuous care retirement communities, senior and subsidised housing, church groups and social service agencies in Chicago and north-eastern Illinois.^21^ At enrolment and annual follow-up visits, participants underwent comprehensive assessments, including a clinical examination, a cognitive test battery and completion of questionnaires on psychological and social well-being.

**Figure 1:**
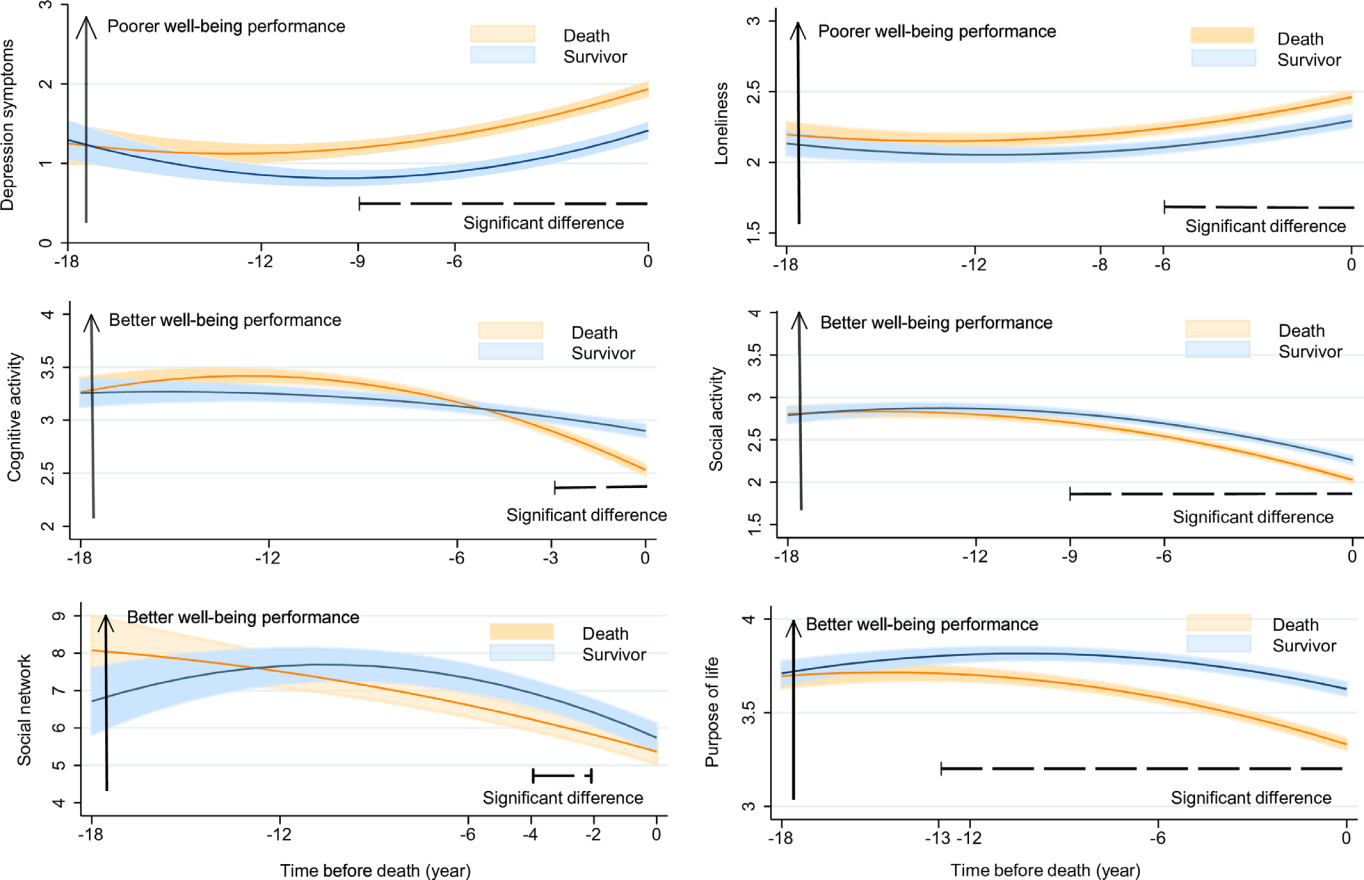
Trajectories of psychological and social well-being by mortality status. Model adjusted for age at time 0 and sex. Significant annual differences between the death and survival groups were corrected by the FDR to avoid false positives.

Selection of the study population is illustrated in online supplemental S Figure 1. A total of 2142 participants underwent a baseline evaluation between 1997 and 2021. From this group, we excluded 271 individuals with missing information on psychological and social well-being at study entry (n=8) and no repeated assessment during the follow-up (n=263), leaving an analytical sample of 1971 participants with at least two times repeated measurements of psychological and social well-being.

### Assessment of psychological and social well-being

The selection of well-being indicators was guided by the goal of capturing key domains of late-life functioning that are theoretically and empirically linked to health outcomes. We intentionally included both self-reported subjective evaluations (eg, purpose in life, loneliness) and more objective metrics (eg, social activity, cognitive activity) to provide a more comprehensive assessment. A total of six dimensions of psychological and social well-being were assessed at baseline and during each annual follow-up examination, including depression symptoms, cognitive activity, social activity, social network, loneliness and purpose in life. The definitions of each domain are presented in online supplemental S Table 1.

*Depression symptoms:* depression symptoms were assessed using a modified, 10-item version of the Center for Epidemiologic Studies Depression Scale. Scores ranged from 0 to 10 and indicate the number of depressive symptoms endorsed.

*Loneliness:* loneliness refers to feeling disconnected from others and was assessed with 5 items from a modified version of the de Jong-Gierveld Loneliness Scale.^22^ Participants were asked to rate their agreement with each item on a 5-point Likert rating scale. The total score ranged from 1 to 5, and higher values indicate more loneliness.

*Cognitive activity:* cognitive activity is a composite measure of the frequency of participation in seven cognitively stimulating activities (including reading, writing letters, visiting a library and playing games such as chess or checkers, etc) during the past year in late life.^23^ Scores ranged from 1 to 5, with higher scores indicating a higher level of cognitive activity.

*Social activity*: the frequency of social activities was assessed using six items that asked participants how often they had participated in common types of activities of social interaction in the past year later in life. Scores for each item ranged from 1 to 5, with higher scores indicating greater social activity.

*Social network size:* social network size was defined as the number of people (eg, children, family and friends) that participants see at least once per month.

*Purpose in life:* purpose in life was measured using 10 items from a modified version of Ryff’s Scales of Psychological Well-Being. Participants were asked to rate their level of agreement with each item on a 5-point scale. Scores ranged from 1 to 5, with higher scores indicating greater purpose in life.

### Mortality

Participants’ survival status was monitored throughout the follow-up period. In addition to annual evaluations, participants are also contacted quarterly to determine survival status, and death is occasionally learnt of during quarterly contacts. Where necessary, we supplemented this by searching death records. At the moment of data analysis, we had accurate death information for almost all study participants.

### Covariates

Covariates were selected based on their established associations with both well-being and mortality in older adults. Information on demographic characteristics (age and sex), socioeconomic status (education and income), lifestyle factors (body mass index (BMI)), alcohol consumption, physical activity), vascular risk factors and cardiovascular disease was collected during the baseline examination (detailed information is provided in online supplemental appendix).

### Statistical analysis

Baseline characteristics of the study population by survival status were compared using χ^2^ tests for categorical variables and the t-test or Wilcoxon rank-sum tests for continuous variables.

*Time-to-event analysis*: Cox proportional hazards models with age as the time scale were used to estimate the HRs) and 95% CIs for mortality in relation to psychological and social well-being. The proportional hazards assumption was tested using Schoenfeld residuals, and no violations were observed. Follow-up time (in years) was calculated as the age from study entry until the age of death or the final examination. Basic-adjusted models included age and sex. Multivariable-adjusted models were further adjusted for education, income, alcohol consumption, physical activity, vascular risk factors and cardiovascular diseases at baseline.

*Retrospective trajectory analysis:* trajectories of psychological and social well-being preceding death were ascertained using a backward timescale, with the year of death (for decedents) or the year corresponding to the end of follow-up (for survivors) set as time 0. This reverse-time approach aligns participants on their endpoint (death or last follow-up), effectively handling the right-censoring of survivors by modelling time until this event. Linear mixed-effects models were used to capture the trajectory of all six domains of psychological and social well-being and the potential differences in these trajectories between survivors and decedents. The fixed effects included survival status, time (year), and/or square of time (year^2^), and their interaction when the corresponding p value <0.05. The random effects included random intercept and slope, allowing individual differences to be reflected at time 0 and across follow-up. Differences in the means and slopes of the overall trajectories of psychological and social well-being between survivors and decedents were estimated, as well as differences at time 0 and at each time point preceding time 0. Detailed information about the model is provided in the online supplemental appendix. To ensure statistical power, trajectory analyses included data on psychological and social well-being up to 18 years preceding death to keep a relatively adequate sample (n>20 for each group) for annual comparison. All analyses were adjusted for age at time 0 and sex. Also, multivariable-adjusted models adjusting for age at time 0, sex, education, income, alcohol consumption, physical activity, vascular risk factors and cardiovascular diseases at baseline and, furthermore, additionally mutually adjusted for indicators of well-being were performed as sensitivity analyses. To better control for the effects of age and sex, we also performed propensity score matching with 1:1 no-return sampling (matching variables were age at time 0 and sex at time 0) as a sensitivity analysis. To explore the sex difference in trajectories of psychological and social well-being preceding death, we conducted sex-stratified analyses. Moreover, we repeated the analyses among participants with more than two repeated measurements of psychological and social well-being.

To further validate the time window for terminal decline, we used a mixed-effects change-point model with piecewise linear trajectories among decedents.^1 24^ The change point was initially selected as the pre-death year with a significant well-being difference between the survival and death groups. The period before the change point was defined as the pre-terminal time, and after the change point was defined as the terminal time. In addition, the slopes of the two time periods were compared (there should be an accelerated decline during the terminal time, ie, there should be a significant statistical difference in the two slopes).

All statistical analyses were performed using Stata SE V.16.0 (Stata Corp, College Station, Texas, USA). P values <0.05 were considered statistically significant. False discovery rate (FDR) correction was applied to all p values to avoid false positives in multiple tests and comparisons of annual differences in psychological and social well-being.

## Findings

### Characteristics of the study population

Among the 1971 study participants, the mean (SD) age was 79.96 (7.42) years at baseline and 87.48 (7.64) years at time 0; 73.47% female. Compared with survivors, decedents were more likely to be older, male, have lower education, income, BMI and levels of alcohol consumption and physical activity and have a higher prevalence of hypertension, stroke, heart disease, claudication and congestive heart failure. Decedents were also more likely to have poorer well-being (table 1). Baseline characteristics of included and excluded subjects generally showed no significant differences (online supplemental S Table 2).

**Table 1:** Characteristics of the study population by survival status at baseline

Characteristics (range)	All (N=1971)	Survivors (N=852)	Decedents (N=1119)	Effect size (95% CI)*	P value
Age at time 0, years (56.60~108.26)	87.48±7.64	83.77±7.70	90.31±6.27	−0.94 (–1.03 to –0.85)	<0.001
Age at baseline, years (53.34~100.47)	79.96±7.42	76.81±7.74	82.36±6.17	−0.81 (–0.90 to –0.72)	<0.001
Female	1448 (73.47)	657 (77.11)	791 (70.69)	0.07 (0.03 to 0.11)	0.001
Education, years (0~30)	15.00±3.31	15.39±3.58	14.71±3.06	0.21 (0.12 to 0.30)	0.001
Income (1~10)	8.00 (5.00, 9.00)	8.00 (6.00, 10.00)	7.00 (5.00, 9.00)	0.31 (0.22 to 0.40)	<0.001
Body mass index, kg/m^2^ (13.94~62.92)	27.33±5.34	27.96±5.67	26.85±5.04	0.21 (0.12 to 0.30)	<0.001
Vascular disease risk factors					
Smoking				0.03 (–0.04 to 0.07)	0.342
Never	1146 (58.26)	484 (56.81)	662 (59.37)		
Ever smoker	770 (39.15)	342 (40.14)	428 (38.39)		
Current smoker	51 (2.59)	26 (3.05)	25 (2.24)		
Diabetes	281 (14.26)	119 (13.97)	162 (14.48)	0.01 (–0.02 to 0.03)	0.749
Hypertension	1561 (79.20)	642 (75.35)	919 (82.13)	0.08 (0.04 to 0.13)	<0.001
Alcohol consumption, g/day (0~234.6)	0.00 (0.00, 5.83)	1.20 (0.00, 7.14)	0.00 (0.00, 5.42)	0.07 (–0.02 to 0.16)	<0.001
Physical activity, hour/week (0~35)	2.50 (0.83, 4.67)	3.00 (1.19, 5.50)	2.25 (0.67, 4.08)	0.27 (0.18 to 0.36)	<0.001
Vascular disease					
Stroke	170 (9.31)	46 (6.02)	124 (11.68)	0.10 (0.06 to 0.13)	<0.001
Heart disease	176 (8.94)	50 (5.88)	126 (11.27)	0.09 (0.06 to 0.13)	<0.001
Claudication	112 (5.69)	37 (4.34)	75 (6.71)	0.05 (–0.00 to 0.11)	0.025
Congestive heart failure	84 (4.68)	27 (3.18)	57 (6.04)	0.07 (0.02 to 0.12)	0.004
Psychological and social well-being at baseline					
Depression symptoms (0~9)	0.00 (0.00, 2.00)	0.00 (0.00, 1.00)	1.00 (0.00, 2.00)	−0.19 (–0.28 to –0.10)	<0.001
Loneliness (1~4.6)	2.25±0.60	2.15±0.59	2.34±0.59	−0.32 (–0.41 to –0.23)	<0.001
Cognitive activity (1~5)	3.18±0.70	3.18±0.63	3.19±0.75	0.01 (–0.10 to 0.08)	0.362
Social Activity (1~4.33)	2.62±0.58	2.74±0.55	2.53±0.58	0.38 (0.29 to 0.47)	<0.001
Social Network (0~46)	7.08±5.87	7.45±6.16	6.80±5.63	0.11 (0.02 to 0.20)	0.012
Purpose in life (2~5)	3.69±0.46	3.81±0.43	3.58±0.46	0.52 (0.42 to 0.61)	<0.001

Values are mean±SD, n (%), or median (IQR).

Missing data: smoking status=4; alcohol consumption=4; body mass index=49; income=205; heart disease=2; claudication=1; stroke=145; depression symptoms=6; cognitive activity=1; social activity=2; social network=12; loneliness=187; purpose in life=189.

* Effect size was calculated by Cohen’s d for continuous variables and Cramér’s V for categorical variables.

### Association of baseline psychological and social well-being with mortality

During the follow-up period (median: 7 years, IQR: 4–11 years), 1119 (56.77%) participants died. In the multivariable-adjusted Cox regressions, depression symptoms and loneliness at baseline were positively associated with increased risk of death, with HRs (95% CIs) of 1.10 (1.06 to 1.14) and 1.28 (1.13 to 1.43), respectively (table 2). In contrast, cognitive activity, social activity, social network and purpose in life were all negatively associated with mortality, with HR (95% CI) of 0.86 (0.78 to 0.94), 0.70 (0.62 0.79), 0.99 (0.97, 1.00) and 0.73 (0.63, 0.86), respectively (table 2). This pattern of results was similar in the basic-adjusted models (table 2). Notably (table 2), when the above six dimensions were further mutually adjusted by incorporating them into the same model, only depression symptoms and social activity were significantly associated with increased mortality, with HRs (95% CIs) of 1.08 (1.03, 1.13) and 0.74 (0.64, 0.85).

**Table 2:** HR and 95% CI for death in relation to psychological and social well-being

Factors	Death					
	HR (95% CI)*	P value	HR (95% CI)†	P value	HR (95% CI)‡	P value
Depression symptoms	1.12 (1.08 to 1.16)	<0.001	1.10 (1.06 to 1.14)	<0.001	1.08 (1.03 to 1.13)	0.001
Loneliness	1.32 (1.18 to 1.47)	<0.001	1.28 (1.13 to 1.43)	<0.001	1.07 (0.93 to 1.23)	0.352
Cognitive activity	0.83 (0.77 to 0.90)	<0.001	0.86 (0.78 to 0.94)	0.002	0.95 (0.85 to 1.06)	0.363
Social activity	0.64 (0.57 to 0.71)	<0.001	0.70 (0.62 to 0.79)	<0.001	0.74 (0.64 to 0.85)	<0.001
Social network	0.99 (0.98 to 1.00)	<0.001	0.99 (0.97 to 1.00)	0.020	1.00 (0.99 to 1.01)	0.704
Purpose in life	0.64 (0.56 to 0.74)	<0.001	0.73 (0.63 to 0.86)	<0.001	0.94 (0.78 to 1.13)	0.477

* Models were adjusted for age and sex.

† Models were adjusted for age, sex, education, income, alcohol consumption, physical activity, vascular risk factors and cardiovascular diseases.

‡ The six dimensions of psychological and social well-being were further mutually adjusted.

### Trajectories of psychological and social well-being preceding death

The included subjects had an average of seven repeated measurements of well-being, ranging from 2 to 19. Figure 1 illustrates the trajectories of psychological and social well-being before death. The severity of depression symptoms and loneliness generally intensified in the years leading up to death. Depression symptoms (β coefficient for difference in rate of change between decedents and survivors: 0.02 (95% CI 0.01 to 0.04), p=0.007) and loneliness (0.01 (0.00 to 0.01), p=0.049) increased faster among decedents than survivors, leading to a significantly higher level of depression symptoms beginning 9 years before death (difference in mean at year −9: 0.35 (0.10 to 0.60)) and a significantly higher level of loneliness 6 years before death (year−6: 0.13 (0.05 to 0.21)) (table 3).

**Table 3:** Differences in psychological and social well-being between survivors and decedents in the 18 years preceding death

Year	No. of survivors	No. of deaths	Depression symptoms	Loneliness	Purpose in life	Cognitive activity	Social activity	Social network
			Difference (95% CI)	Difference (95% CI)	Difference (95% CI)	Difference (95% CI)	Difference (95% CI)	Difference (95% CI)
−18	39	25	−0.40 (−1.58 to 0.77)	0.07 (−0.37 to 0.52)	−0.29 (−0.62 to 0.04)	−0.01 (−0.49 to 0.46)	−0.12 (−0.47 to 0.25)	1.14 (−0.17 to 3.97)
−17	71	38	0.05 (−0.63 to 0.74)	0.01 (−0.29 to 0.32)	−0.22 (−0.44 to 0.01)	−0.03 (−0.30 to 0.25)	−0.25 (−0.50 to 0.01)	−0.95 (−4.54 to 2.63)
−16	89	52	0.46 (−0.08 to 1.00)	0.03 (−0.24 to 0.29)	−0.11 (−0.29 to 0.08)	0.06 (−0.18 to 0.31)	−0.02 (−0.22 to 0.18)	−1.16 (−3.92 to 0.75)
−15	103	72	0.22 (−0.30 to 0.74)	0.23 (−0.02 to 0.48)	−0.06 (−0.22 to 0.11)	−0.03 (−0.24 to 0.19)	−0.17 (−0.36 to 0.01)	−0.98 (−3.08 to 1.11)
−14	110	96	−0.01 (−0.46 to 0.44)	0.04 (−0.18 to 0.25)	−0.03 (−0.18 to 0.12)	0.11 (−0.08 to 0.30)	−0.07 (−0.23 to 0.10)	−0.57 (−2.60 to 1.46)
−13	134	146	0.34 (−0.06 to 0.74)	0.02 (−0.15 to 0.19)	−0.14 (−0.26 to −0.01)	0.04 (−0.12 to 0.20)	−0.08 (−0.22 to 0.07)	0.61 (−1.01 to 2.23)
−12	169	192	0.04 (−0.29 to 0.38)	−0.01 (−0.16 to 0.14)	−0.12 (−0.23 to −0.01)	0.05 (−0.10 to 0.20)	−0.08 (−0.20 to 0.05)	1.17 (−0.18 to 2.51)
−11	209	246	0.34 (0.01 to 0.66)	0.04 (−0.08 to 0.17)	−0.16 (−0.26 to −0.06)	0.07 (−0.07 to 0.20)	−0.13 (−0.25 to −0.02)	0.28 (−0.10 to 1.56)
−10	248	300	0.22 (−0.05 to 0.49)	0.07 (−0.05 to 0.19)	−0.07 (−0.16 to −0.02)	0.08 (−0.05 to 0.21)	−0.08 (−0.18 to 0.03)	0.37 (−0.74 to 1.49)
−9	304	369	0.35 (0.10 to 0.60)	0.11 (0.00 to 0.21)	−0.15 (−0.23 to −0.07)	−0.01 (−0.12 to 0.12)	−0.16 (−0.26 to −0.06)	−0.78 (−1.72 to 0.17)
−8	335	456	0.53 (0.28 to 0.77)	0.15 (0.05 to 0.25)	−0.18 (−0.26 to −0.11)	−0.01 (−0.12 to 0.11)	−0.17 (−0.26 to −0.07)	−0.66 (−1.57 to 0.25)
−7	379	535	0.42 (0.19 to 0.65)	0.09 (−0.00 to 0.17)	−0.16 (−0.23 to −0.09)	0.01 (−0.10 to 0.12)	−0.14 (−0.23 to −0.06)	−0.77 (−1.68 to 0.14)
−6	427	632	0.51 (0.28 to 0.74)	0.13 (0.05 to 0.21)	−0.17 (−0.24 to −0.11)	−0.04 (−0.14 to 0.06)	−0.22 (−0.30 to −0.14)	−0.80 (−1.60 to 0.00)
−5	486	691	0.49 (0.29 to 0.70)	0.14 (0.05 to 0.22)	−0.20 (−0.27 to −0.14)	−0.05 (−0.15 to 0.04)	−0.22 (−0.29 to −0.14)	−0.88 (−1.62 to −0.14)
−4	608	785	0.48 (0.28 to 0.68)	0.15 (0.08 to 0.23)	−0.19 (−0.25 to −0.13)	−0.04 (−0.13 to 0.05)	−0.20 (−0.27 to −0.13)	−1.06 (−1.77 to −0.36)
−3	685	841	0.36 (0.18 to 0.54)	0.18 (0.10 to 0.25)	−0.25 (−0.30 to −0.19)	−0.12 (−0.21 to −0.04)	−0.25 (−0.32 to −0.18)	−0.95 (−1.60 to −0.30)
−2	738	936	0.37 (0.18 to 0.56)	0.17 (0.10 to 0.24)	−0.29 (−0.35 to −0.24)	−0.15 (−0.23 to −0.06)	−0.25 (−0.32 to −0.18)	−1.06 (−1.65 to −0.46)
−1	728	970	0.42 (0.23 to 0.62)	0.08 (0.01 to 0.15)	−0.26 (−0.31 to −0.20)	−0.26 (−0.35 to −0.17)	−0.09 (−0.16 to −0.02)	−0.35 (−0.91 to 0.21)
0	820	967	0.39 (0.19 to 0.60)	0.10 (0.03 to 0.18)	−0.25 (−0.31 to −0.19)	−0.32 (−0.40 to −0.23)	−0.18 (−0.25 to −0.12)	0.03 (−0.52 to 0.59)
Annual change			0.12 (0.10 to 0.13)	0.04 (0.04 to 0.05)	−0.04 (−0.04 to −0.03)	−0.05 (−0.06 to −0.04)	−0.09 (−0.10 to −0.09)	−0.37 (−0.44 to −0.30)
Difference in mean			0.56 (0.41 to 0.71)	0.17 (0.11 to 0.22)	−0.30 (−0.34 to −0.25)	−0.37 (−0.44 to −0.29)	−0.24 (−0.29 to −0.18)	−0.37 (−0.86 to 0.12)
Difference in change			0.02 (0.01 to 0.04)	0.01 (0.00 to 0.01)	−0.02 (−0.02 to −0.01)	−0.09 (−0.10 to −0.08)	−0.01 (−0.02 to −0.01)	0.01 (0.01 to 0.02)

The difference was calculated as the mean of each measure in the death group minus that in the survivor group. The difference in change means the fixed effect of the interaction between death and time. Models were adjusted for age at time 0 and sex. P values were FDR-corrected as q values. Bolded text indicates statistically significant after correction.

Cognitive activity, social activity, social network and purpose in life all declined preceding death (figure 1). compared with survivors, decedents showed a faster decline in cognitive activity, social activity and purpose in life (table 3), resulting in significantly lower levels of purpose in life beginning 13 years before death (difference in mean at year-13: −0.14 (–0.26,–0.01)), social activity beginning 9 years before death (year −9: −0.16 (–0.26,–0.06)), and cognitive activity beginning 3 years before death (year −3: −0.12 (-0.21,–0.04)). Trajectories in social network size between the decedents and survivors intersected over the follow-up such that decedents had a larger social network initially, but it became significantly lower than that of the survivors during the 2 to 4 years preceding death (year −4 and −2: -0.14 (-0.26,–0.01) and -1.06 (−1.65 to –0.46)). The random effects of all these models are shown in online supplemental S Table 3, and the results indicate significant individual differences in all well-being indicators in terms of initial status (intercept) and rate of change (slope).

The trajectories of psychological and social well-being preceding death were similar after multi-variable adjustments (online supplemental S Table 4), mutually adjusted for well-being indicators (online supplemental S Table 5), and after excluding participants with less than 3 repeated measures of psychological and social well-being (online supplemental S Table 6 and S Figure 2), and after matching (online supplemental S Table 7). As an exploratory step, we generated two composite scores for psychological well-being (depressive symptoms, loneliness and purpose in life) and social well-being (cognitive activity, social activity, and social network). Significant differences in psychological well-being began 9 years before death and social well-being began 6 years before death among survivors and decedents (online supplemental S Table 8).

In sex-stratified analyses, the severity of both depressive symptoms and loneliness was significantly higher in female decedents (compared with female survivors) beginning 9 years before death; in contrast, depressive symptoms were higher in male decedents (compared with male survivors) beginning 6 years before death and 3 years before death for loneliness (online supplemental S Figure 3 and S Tables 9 and 10). Significantly lower cognitive activity, social activity and purpose in life were observed from 3 years, 9 years and 11 years before death for female decedents and from 1 year, 8 years and 8 years for male decedents, respectively (online supplemental S Tables 9 and 10). Significant differences in social networks between decedents and survivors emerged 4–2 years before death for women and 8–5 years for men. There was a significant interaction between sex and survival status on the decline in the purpose in life (p value for interaction=0.045), but not on the changes in other domains of psychological and social well-being.

Among decedents (online supplemental S Table 11 and S Figure 4), the terminal changes in purpose in life began 11 years before death (difference in slope before and after change point: −0.02 (–0.03,–0.01); the slope of decline accelerates twofold in the terminal phase than pre-terminal phase), compared with 10 years before death for cognitive activity (−0.06 (–0.09,–0.04); accelerates threefold), 9 years for social activity (−0.05 (-0.06,–0.04); accelerates twofold) and depression symptoms (0.08 (0.04, 0.13); accelerates eightfold) and 6 years for loneliness (0.02 (0.01, 0.03); accelerates fourfold). No terminal decline was observed for social network size (online supplemental S Table 11).

## Discussion

In this community-based cohort study of older adults, we explored how psychological and social well-being indicators change in the years preceding death. We found that (1) high depression symptoms and poor social activity were associated with increased mortality; (2) psychological and social well-being declined faster among decedents compared with survivors; and (3) terminal deteriorations were observed beginning 9 years preceding death for depression symptoms and social activity, 6 years for loneliness, 10 years for cognitive activity and even 11 years for purpose in life.

Our results align with previous research reporting significant associations between poorer psychological and social well-being indices, including depressive symptoms,^4^ loneliness,^5^ purpose in life,^6^ social activity^7 8^ and social network size,^9^ and higher mortality. Recent meta-analyses have linked loneliness and smaller social network size to a higher risk of death.^5 9^ In contrast, other cohort studies reported negative findings.^11 12^ For instance, in a subsample of the Midlife in the United States National Study (n=3975), there was no significant association between a higher frequency of loneliness and mortality.^11^ Another study from the Panel on Health and Ageing of Singaporean Elderly indicated that women who experience loneliness have an elevated risk of death, but men do not, and depressive symptoms are not related to increased mortality risk for either women or men.^12^ In our study, all six measures of psychological and social well-being were preliminarily associated with mortality, whereas, after indepth adjustment, only depressive symptoms and social activity remained as predictors of death. The independent associations of depressive symptoms and social activity with mortality may indicate that they are sensitive proxies of yet unmeasured deteriorations, such as subclinical functioning decline or decreasing motivational drive, which are directly linked to the dying process. These findings suggest that baseline well-being, especially depressive symptoms and social activity, may help identify elderly people at risk of premature death.

Dynamic fluctuations in physiological and psychological states during the pre-terminal phase are a distinctive feature of the end-of-life process.^25^ The non-linear deterioration of multi-system functions at this stage increases the difficulty of end-of-life care.^25^ In particular, the evolutionary pattern of psychological and social well-being at the end of life is controversial, with the direction (improves or declines), rate of change over time and key turning points undefined, and systematic evidence for key time windows prior to death is largely unknown. A longitudinal study of 2910 participants revealed a significant terminal decline in life satisfaction in the years before death, and this was associated with a significant worsening of social well-being and social goals.^26^ Findings from 2411 participants (age: 29–95 years) from the Interplay of Genes and Environments Across Multiple Studies revealed accelerated increases in depressive symptoms approximately 4 years before death.^20^

Our study expands on this literature through the use of retrospective trajectory analyses, anchored to the date of death. This enabled us to map the specific trajectories of psychological and social well-being before death and identify the time window during which terminal declines in these domains began. We found that the trajectory of different psychological and social well-being indicators was varied, with a significant difference in purpose in life between decedents and survivors occurring as early as 13 years before death and differences in cognitive activity occurring 3 years before death. The differential timing of terminal decline across well-being domains may reflect a pattern of hierarchical vulnerability, where psychologically complex resources, such as purpose in life, diminish earlier than more externally supported or behavioural activities, like cognitive activity, suggesting their heightened sensitivity to underlying subclinical pathological processes.

In addition, as early as 11 years before death, we observed an accelerated decline in purpose in life among decedents (ie, terminal decline). Our findings indicate that purpose in life may be more psychologically demanding than other domains of psychological and social well-being, and therefore may serve as an especially sensitive indicator of mortality. In addition, women are more likely to experience a longer period of psychological terminal decline than men, especially in the domains of loneliness and purpose in life. Our analysis suggests that individual psychological and social well-being trajectory heterogeneity is meaningfully associated with mortality. In particular, we found an intersection in the trajectories of social network size between decedents and survivors whereby the decedents initially have larger social networks but have significantly smaller social networks than the survivors in the 2–4 years prior to death. This may be the reason why the association between social network and death in previous studies has been inconsistent and the traditional cohort design and time-to-event analysis cannot accurately identify the risk effects of this time-dependent variable.

Mortality-related functioning loss might be the leading cause of psychological and social well-being terminal decline observed in this study.^16^ Factors such as frailty, sensory loss (ie, visual and hearing impairments) and multimorbidity are common in older adults, and these are all associated with an accelerated decline in psychological and social well-being.^2^ Moreover, in late life, the body will experience changes in hormonal status and the accumulation of inflammation and oxidative stress,^27^ which could also accelerate the emergence of psychological and social well-being terminal decline.^28^ Among them, the fluctuation of hormone levels may be related to sex-specific terminal decline.^27^ In particular, women experience large fluctuations in hormone levels after menopause, which is not only related to changes in their physiological function (which in turn influences well-being) but may also be directly related to psychological ageing.^29^ There is also evidence that males and females have different coping strategies in the face of challenging events, and this may render women especially vulnerable to developing declines in psychological and social well-being.

Strengths of this study include the community-based cohort design, large sample size and long-term annual follow-up. In addition, our study used a novel approach based on long-term trajectories to characterise the terminal decline of multidimensional psychological and social well-being in the lead-up to death. Nonetheless, some limitations should be pointed out. First, as volunteers, the MAP participants were generally healthier and more highly educated than the general population, and these characteristics are associated with lower mortality.^30^ This may leave open the possibility that our results might underestimate the complex association between well-being and mortality. Therefore, caution is needed when our findings are generalised to other populations. Second, due to limited statistical power, we could not separately assess psychological and social well-being trajectories in relation to specific causes of death. That said, we suspect that the patterns of terminal decline for specific causes of death (cancer, suicide, etc) might be different, and future studies with larger samples are warranted to further explore this question. Third, given our preference for some objective indicators of social and psychological well-being, we did not include the other five dimensions beyond Ryff scales on purpose in life. Fourth, the death time for individuals who survived at time 0 is unclear, which may lead to conservative estimates of terminal decline, as individuals who died shortly after their last follow-up would contribute pre-terminal rather than terminal phase data. However, change-point modelling conducted in the death sample supports the terminal decline in well-being. Fifth, the statistical model used in this study has limitations in accounting for individual differences in the terminal decline time window, and future studies could use alternative models that can accurately estimate random turning points. Finally, although we accounted for a wide range of covariates in our analyses, we cannot rule out the possibility of residual confounding due to unmeasured factors, especially the time-varying confounding factors.

### Clinical implications

The study provides insights into the terminal downward trajectory of psychological and social well-being at the end of life and their complex associations with death. We found that significant declines in psychological and social well-being began as early as 11 years before death. These results suggest that strategies to delay declines in psychological and social well-being should be implemented while decades of life expectancy remain. Early detection of changes in psychological and social well-being—especially purpose in life—may provide opportunities for targeted interventions to prolong healthy longevity. Future studies could elucidate the terminal deafferentation patterns of well-being to indicate how correlations and differences in terminal decline across different indicators of well-being.

## Supplementary material

10.1136/bmjment-2025-301967 online supplemental file 1

## Data Availability

Data may be obtained from a third party and are not publicly available.
