# Supplementary material for: Trajectories of psychological and social well-being preceding death
Source: BMJ Ment Health. 2025 Nov 7;28(1):e301967. doi: 10.1136/bmjment-2025-301967 (PMC12599000; doi:10.1136/bmjment-2025-301967)
Supplement: online supplemental file 1 [file bmjment-28-1-s001.docx]

**Data collection**

Information on demographic characteristics, socioeconomic status, and lifestyle factors was collected at baseline ^1^. Education was quantified as the number of years of regular schooling. Income was determined by the "show card" method in which subjects were asked to select one of 10 total family income levels, with scores ranging from 1 to 10, and 10 denoting the highest income level^2^. Body mass index (BMI) was calculated by weight (kg)/height (m^2^). Alcohol consumption was measured by the average amount of alcohol (in grams) consumed per day over the past year. Physical activity was assessed using questions adapted from the National Health Interview Survey ^3^. Physical activity was defined as the sum of hours per week that the participant engaged in 5 activities (i.e., gardening or yard work, walking for exercise, bicycle riding, calisthenics or general exercise, and swimming or water exercise) within the past 2 weeks, with questions adapted from the National Health Interview Survey ^4^. Vascular disease risk factors included smoking (never, ever, and current smoker), hypertension, and diabetes mellitus ^5^. Vascular diseases, including stroke, heart disease (i.e., heart attack or coronary, myocardial infarction, coronary occlusion, or coronary thrombosis), claudication, and congestive heart failure, were derived from the baseline medical history and clinical evaluation ^6^.

**Reference**

1. Bennett DA, Schneider JA, Buchman AS, Barnes LL, Boyle PA, Wilson RS. Overview and findings from the rush Memory and Aging Project. *Current Alzheimer research*. Jul 2012;9(6):646-63. doi:10.2174/156720512801322663

2. Bennett DA, Schneider JA, Buchman AS, Mendes de Leon C, Bienias JL, Wilson RS. The Rush Memory and Aging Project: study design and baseline characteristics of the study cohort. *Neuroepidemiology*. 2005;25(4):163-75. doi:10.1159/000087446

3. Song R, Xu H, Dintica CS, et al. Associations Between Cardiovascular Risk, Structural Brain Changes, and Cognitive Decline. *Journal of the American College of Cardiology*. May 26 2020;75(20):2525-2534. doi:10.1016/j.jacc.2020.03.053

4. Krueger KR, Wilson RS, Shah RC, Tang Y, Bennett DA. Personality and incident disability in older persons. *Age Ageing*. Jul 2006;35(4):428-33. doi:10.1093/ageing/afl028

5. Boyle PA, Wilson RS, Aggarwal NT, Tang Y, Bennett DA. Mild cognitive impairment: risk of Alzheimer disease and rate of cognitive decline. *Neurology*. Aug 8 2006;67(3):441-5. doi:10.1212/01.wnl.0000228244.10416.20

6. Boyle PA, Wilson RS, Aggarwal NT, et al. Parkinsonian signs in subjects with mild cognitive impairment. *Neurology*. Dec 27 2005;65(12):1901-6. doi:10.1212/01.wnl.0000188878.81385.73

**Mixed-effects model**

The linear mixed-effects model used in the analyses of the trajectories of social and psychological well-being before death is as follows.

$$y=\beta_{0}+\beta_{1}time+\beta_{2}{time}^{2}+\beta_{3}death+\beta_{4}death*time+\beta_{5}covariates+Z\gamma+ɛ$$

- y is the well-being
- time is aligned at the year of death for decedents and at the end of follow-up for survivors
- death is a binary variable (Yes vs. No)
- Z is the known matrix of random effects
- γ is the unknown vector of random-effects parameters
- ε is the unobserved vector of random errors.

The piecewise mixed-effects change-point model used in the analyses of the trajectories of social and psychological well-being before death among decedents is as follows.

$$y=\beta_{0}+\beta_{1}time+\beta_{2}change-point+\beta_{3}change-point*time+\beta_{4}covariates+Z\gamma+ɛ$$

- y is the well-being
- time is aligned at the year of death
- change-point is a binary variable (Before vs. After)
- Z is the known matrix of random effects
- γ is the unknown vector of random-effects parameters
- ε is the unobserved vector of random errors.

Baseline participants, n=2,242

We excluded 271 participants due to missing psychological and social well-being data at baseline (n=8) or missing follow-up data (n=263)

Analytical sample, n=1,971

Survival at endpoint, n=852

Death at endpoint, n=1,119

**S Figure 1.** Flowchart of the study population.

**S Table 1.** Definition of psychological and social well-being

| Domains | Item | Score |
| --- | --- | --- |
| *Depression symptom* | | |
| 1 | I felt depressed. | Yes=1 |
| 2 | I felt that everything I did was an effort. | Yes=1 |
| 3 | My sleep was restless. | Yes=1 |
| 4 | I was happy. | No=1 |
| 5 | I felt lonely. | Yes=1 |
| 6 | People were unfriendly. | Yes=1 |
| 7 | I enjoyed life. | No=1 |
| 8 | I felt sad. | Yes=1 |
| 9 | I felt that people disliked me. | Yes=1 |
| 10 | I could not get going. | Yes=1 |
| *Cognitive activity* | | |
| 1 | About how much time do you spend reading each day? | Item 1 \| 2-7:  1 None \| Once a year  2 Less than one hour \| Several times a year  3 One to less than two hours \| Several times a month  4 Two to less than three hours \| Several times a week  5 Three or more hours \| Every day/almost every day  *Item scores are averaged to yield the composite measure.* |
| 2 | In the last year, how often did you visit a library? |  |
| 3 | Thinking of the last year, how often do you read newspapers? |  |
| 4 | During the past year, how often did you read magazines? |  |
| 5 | During the past year, how often did you read books? |  |
| 6 | During the past year, how often did you write letters? |  |
| 7 | During the past year, how often did you play games like checkers or other board games, cards, puzzles, etc.? |  |
| Social activity | | |
| During the past year, how often did you...   1. go to restaurants, sporting events or teletract, or play bingo? 2. go on day trips or overnight trips? 3. do unpaid community/volunteer work? 4. visit at relatives' or friends' houses? 5. participate in groups (such as senior center, VFW, Knights of Columbus, Rosary Society, or something similar)? 6. attend church or religious services? | | 1 Once a year or less  2 Several times a year  3 Several times a month  4 Several times a week  5 Every day or almost every day  *Item scores are averaged to yield the composite measure.* |
| *Social network* | | |
| 1. How many living children do you have? 2. How many of your children do you see at least once a month? 3. Do you see your child at least once a month? 4. How many close relatives do you have? 5. How many of these close relatives do you usually see at least once a month? 6. Do you see your close relative at least once a month? 7. How many close friends do you have? 8. How many of these close friends do you see at least once a month? 9. Do you see your close friend at least once a month? | | Sum the number of these individuals (children, family, and friends) seen at least once a month. |
| *Loneliness* | | |
| Participants are presented with the following items:   1. I experience a general sense of emptiness. 2. I miss having people around. 3. I feel like I don't have enough friends. 4. I often feel abandoned. 5. I miss having a really close friend. | | 1 Strongly disagree  2 Disagree  3 Neutral  4 Agree  5 Strongly agree  *Item scores are averaged to yield the composite measure.* |
| *Purpose in life* | | |
| Participants are asked the following items:   1. I feel good when I think of what I've done in the past and what I hope to do in the future. 2. I live life one day at a time and don't really think about the future. 3. I tend to focus on the present, because the future nearly always brings me problems. 4. I have a sense of direction and purpose in life. 5. My daily activities often seem trivial and unimportant to me. 6. I used to set goals for myself, but that now seems like a waste of time. 7. I enjoy making plans for the future and working them to a reality. 8. I am an active person in carrying out the plans I set for myself. 9. Some people wander aimlessly through life, but I am not one of them. 10. I sometimes feel as if I've done all there is to do in life. | | Item 1,4,7-9 \| 2-3,5-6,10:  1 Strongly disagree  2 Disagree  3 Neutral  4 Agree  5 Strongly agree  *Item scores are averaged to yield the composite measure.* |

**S Table 2.** Characteristics of included and excluded populations.

| **Characteristics** | **Included  (N=1971)** | **Excluded (N=271)** | ***P-value*** |
| --- | --- | --- | --- |
| Age at baseline, yrs | 79.96 ± 7.42 | 800.73 ± 8.57 | 0.115 |
| Female | 1,448 (73.47) | 195 (71.96) | 0.598 |
| Education, yrs | 15.00 ± 3.31 | 14.42 ± 3.53 | 0.1146 |
| Income | 8.00 (5.00, 9.00) | 7.00 (5.00, 9.00) | 0.605 |
| Body mass index, kg/m^2^ | 27.33 ± 5.34 | 27.16 ± 5.777 | 0.633 |
| Vascular disease risk factors |  |  |  |
| Smoking |  |  | 0.451 |
| Never | 1,146 (58.26) | 142 (54.20) |  |
| Ever smoker | 770 (39.15) | 113 (43.13) |  |
| Current smoker | 51 (2.59) | 7 (2.67) |  |
| Diabetes | 281(14.26) | 28 (10.33) | 0.883 |
| Hypertension | 1,561 (79.20) | 198 (73.33) | 0.836 |
| Alcohol consumption, g/day | 0.00 (0.00, 5.83) | 0.00 (0.00, 4.31) | 0.127 |
| Physical activity, h/week | 2.50 (0.83, 4.67) | 2.08 (0.43, 4.40) | 0.115 |
| Vascular disease |  |  |  |
| Stroke | 170 (9.31) | 20 (8.33) | 0.623 |
| Heart disease | 176 (8.94) | 24 (8.89) | 0.979 |
| Claudication | 112 (5.69) | 16 (6.02) | 0.828 |
| Congestive heart failure | 84 (4.68) | 22 (9.40) | 0.002 |
| Psychological and social well-being at baseline |  |  |  |
| Depression symptoms | 0.00 (0.00, 2.00) | 1.00 (0.00, 2.00) | 0.244 |
| Loneliness | 2.25 ± 0.60 | 2.31 ± 0.62 | 0.116 |
| Cognitive activity | 3.18 ± 0.70 | 3.16 ± 0.78 | 0.554 |
| Social Activity | 2.62 ± 0.58 | 2.47 ± 0.61 | <0.001 |
| Social Network | 7.08 ± 5.87 | 7.04 ± 5.41 | 0.925 |
| Purpose in life | 3.69 ± 0.46 | 3.59 ± 0.46 | 0.060 |

Values are mean ± SD, n (%), or median (interquartile range).

**S Table 3.** Random effects of mixed-effects models.

| **Models** | **Parameters** | **Depression symptoms** | **Loneliness** | **Cognitive activity** | **Social Activity** | **Social Network** | **Purpose of life** |
| --- | --- | --- | --- | --- | --- | --- | --- |
|  |  | Difference (95% CI) | Difference (95% CI) | Difference (95% CI) | Difference (95% CI) | Difference (95% CI) | Difference (95% CI) |
| Null model | var(year) | **0.011 (0009, 0.014)** | **0.001 (0.001, 0.002)** | **0.009 (0.008, 0.010)** | **0.006 (0.005, 0.006)** | **0.087 (0.070, 0.108)** | **0.001 (0.001, 0.001)** |
|  | var(_cons) | **2.086 (1.924, 2.261)** | **0.289 (0.267, 0.312)** | **0.628 (0.585, 0.674)** | **0.359 (0.331, 0.388)** | **17.477 (16.134, 18.931)** | **0.231 (0.215, 0.248)** |
|  | cov(year,_cons) | **0.098 (0.081, 0.115)** | **0.009 (0.007, 0.011)** | **0.051 (0.045, 0.056)** | **0.030 (0.026, 0.033)** | **0.227 (0.103, 0.350)** | **0.010 (0.008, 0.011)** |
| Full model | var(year) | **0.008 (0.007, 0.010)** | **0.001 (0.001, 0.001)** | **0.004 (0.003, 0.005)** | **0.001 (0.001, 0.002)** | **0.063 (0.049, 0.080)** | **0.001 (0.001, 0.001)** |
|  | var(_cons) | **1.980 (1.828, 2.145)** | **0.273 (0.253, 0.295)** | **0.551 (0.515, 0.590)** | **0.284 (0.264, 0.306)** | **17.298 (15.982, 18.721)** | **0.192 (0.179, 0.201)** |
|  | cov(year,_cons) | **0.083 (0.068, 0.098)** | **0.007 (0.005, 0.009)** | **0.029 (0.026, 0.033)** | **0.012 (0.010, 0.014)** | **0.151 (0.043, 0.260)** | **0.006 (0.005, 0.007)** |

Full models were adjusted for age at time 0 and sex.

**S Table 4.** Differences in psychological and social well-being between survivors and decedents in the 18 years preceding death.

|  | **Depression symptoms** | | **Loneliness** | | **Cognitive activity** | | **Social Activity** | | **Social Network** | | **Purpose of life** | |
| --- | --- | --- | --- | --- | --- | --- | --- | --- | --- | --- | --- | --- |
|  | Difference (95% CI) | *P* | Difference (95% CI) | *P* | Difference (95% CI) | *P* | Difference (95% CI) | *P* | Difference (95% CI) | *P* | Difference (95% CI) | *P* |
| Annual change | **0.12 (0.10, 0.14)** | **<0.001** | **0.04 (0.04, 0.05)** | **<0.001** | **-0.05 (-0.06, -0.04)** | **<0.001** | **-0.09 (-0.10, -0.09)** | **<0.001** | **-0.38 (-0.45, -0.30)** | **<0.001** | **-0.04 (-0.04, -0.03)** | **<0.001** |
| Difference in mean | **0.35 (0.20, 0.51)** | **<0.001** | **0.11 (0.05, 0.16)** | **<0.001** | **-0.28 (-0.35, -0.20)** | **<0.001** | **-0.16 (-0.22, -0.10)** | **<0.001** | -0.20 (-0.72, 0.33) | 0.460 | **-0.23 (-0.27, -0.18)** | **<0.001** |
| Difference in change | 0.01 (-0.00, 0.03) | 0.09 | 0.00 (-0.00, 0.01) | 0.121 | **-0.09 (-0.10, -0.08)** | **<0.001** | **-0.01 (-0.02, -0.01)** | **<0.001** | **0.01 (0.01, 0.02)** | **0.001** | **-0.01 (-0.02, -0.01)** | **<0.001** |

Difference was calculated as the mean of each measure in the death group minus that in the survivor group. Difference in change means the fixed effect of interaction between death and time. Models were adjusted for age at time 0, sex, education, income, alcohol consumption, physical activity, vascular risk factors, and cardiovascular diseases.

**S Table 5.** Differences in psychological and social well-being between survivors and decedents in the 18 years preceding death.

|  | **Depression symptoms** | | **Loneliness** | | **Cognitive activity** | | **Social Activity** | | **Social Network** | | **Purpose of life** | |
| --- | --- | --- | --- | --- | --- | --- | --- | --- | --- | --- | --- | --- |
|  | Difference (95% CI) | *P* | Difference (95% CI) | *P* | Difference (95% CI) | *P* | Difference (95% CI) | *P* | Difference (95% CI) | *P* | Difference (95% CI) | *P* |
| Annual change | **0.20 (0.07, 0.33)** | **0.002** | **0.01 (0.01, 0.02)** | **<0.001** | **-0.02 (-0.03, -0.01)** | **<0.001** | **-0.06 (-0.07, -0.06)** | **<0.001** | **-0.23 (-0.30, -0.15)** | **<0.001** | **-0.01 (-0.02, -0.01)** | **<0.001** |
| Difference in mean | **0.06 (0.05, 0.08)** | **<0.001** | 0.01 (-0.05, 0.04) | 0.838 | **-0.21 (-0.28, -0.14)** | **<0.001** | **-0.10 (-0.15, -0.06)** | **<0.001** | 0.07 (-0.40, 0.55) | 0.764 | **-0.21 (-0.25, -0.18)** | **<0.001** |
| Difference in change | -0.01 (-0.02, 0.01) | 0.849 | 0.00 (-0.01, 0.01) | 0.355 | **-0.06 (-0.07, -0.05)** | **<0.001** | -0.01 (-0.01, 0.01) | 0.311 | **0.17 (0.06, 0.28)** | **0.001** | **-0.01 (-0.02, -0.01)** | **<0.001** |

Difference was calculated as the mean of each measure in the death group minus that in the survivor group. Difference in change means the fixed effect of interaction between death and time. Models were adjusted for age at time 0 and sex, and mutually adjusted for well-being indicators.

**S Table 6.** Differences in psychological and social well-being between survivors and decedents in the 18 years preceding death (with ≥3 repeated measurements).

| **Year** | **No. of survivor** | **No. of death** | **Depression symptoms** | | **Cognitive activity** | | **Social Activity** | | **Social Network** | | **Loneliness** | | **Purpose of life** | |
| --- | --- | --- | --- | --- | --- | --- | --- | --- | --- | --- | --- | --- | --- | --- |
|  |  |  | Difference (95% CI) | *P* | Difference (95% CI) | *P* | Difference (95% CI) | *P* | Difference (95% CI) | *P* | Difference (95% CI) | *P* | Difference (95% CI) | *P* |
| -18 | 39 | 25 | -0.40 (-1.57, 0.78) | 0.603 | -0.01 (-0.49, 0.46) | 0.964 | -0.12 (-0.48, 0.25) | 0.559 | 1.14 (-0.17, 3.97) | 0.626 | 0.07 (-0.37, 0.52) | 0.927 | -0.29 (-0.62, 0.04) | 0.107 |
| -17 | 71 | 38 | 0.05 (-0.63, 0.74) | 0.925 | -0.03 (-0.30, 0.25) | 0.964 | -0.25 (-0.50, 0.01) | 0.087 | -0.95 (-4.54, 2.63) | 0.674 | 0.01 (-0.29, 0.32) | 0.927 | -0.22 (-0.44, 0.01) | 0.079 |
| -16 | 89 | 52 | 0.46 (-0.08, 1.00) | 0.142 | 0.06 (-0.18, 0.31) | 0.908 | -0.02 (-0.22, 0.18) | 0.843 | -1.16 (-3.92, 0.75) | 0.314 | 0.03 (-0.24, 0.29) | 0.927 | -0.11 (-0.29, 0.08) | 0.294 |
| -15 | 103 | 72 | 0.22 (-0.30, 0.74) | 0.509 | -0.03 (-0.24, 0.19) | 0.964 | -0.17 (-0.36, 0.01) | 0.104 | -0.98 (-3.08, 1.11) | 0.565 | 0.23 (-0.02, 0.48) | 0.133 | -0.06 (-0.22, 0.11) | 0.530 |
| -14 | 110 | 96 | -0.01 (-0.46, 0.44) | 0.965 | 0.11 (-0.08, 0.30) | 0.676 | -0.07 (-0.23, 0.10) | 0.466 | -0.57 (-2.60, 1.46) | 0.674 | 0.04 (-0.18, 0.25) | 0.927 | -0.03 (-0.18, 0.12) | 0.676 |
| -13 | 134 | 146 | 0.34 (-0.06, 0.74) | 0.142 | 0.04 (-0.12, 0.20) | 0.908 | -0.08 (-0.22, 0.07) | 0.348 | 0.61 (-1.01, 2.23) | 0.631 | 0.02 (-0.15, 0.19) | 0.927 | **-0.14 (-0.26, -0.01)** | **0.045** |
| -12 | 169 | 192 | 0.04 (-0.29, 0.38) | 0.893 | 0.05 (-0.10, 0.20) | 0.908 | -0.08 (-0.20, 0.05) | 0.308 | 1.17 (-0.18, 2.51) | 0.239 | -0.01 (-0.16, 0.14) | 0.927 | **-0.12 (-0.23, -0.01)** | **0.038** |
| -11 | 209 | 246 | 0.34 (0.01, 0.66) | 0.071 | 0.07 (-0.07, 0.20) | 0.724 | -0.13 (-0.25, -0.02) | 0.055 | 0.28 (-0.10, 1.56) | 0.714 | 0.04 (-0.08, 0.17) | 0.721 | **-0.16 (-0.26, -0.06)** | **0.003** |
| -10 | 248 | 300 | 0.22 (-0.05, 0.49) | 0.148 | 0.08 (-0.05, 0.21) | 0.676 | -0.08 (-0.18, 0.03) | 0.214 | 0.37 (-0.74, 1.49) | 0.649 | 0.07 (-0.05, 0.19) | 0.415 | **-0.07 (-0.16, -0.02)** | **0.006** |
| -9 | 304 | 369 | **0.35 (0.10, 0.60)** | **0.013** | -0.01 (-0.12, 0.12) | 0.964 | **-0.16 (-0.26, -0.06)** | **0.004** | -0.78 (-1.72, 0.17) | 0.239 | 0.11 (0.00, 0.21) | 0.105 | **-0.15 (-0.23, -0.07)** | **<0.001** |
| -8 | 335 | 456 | **0.53 (0.28, 0.77)** | **<0.001** | -0.01 (-0.12, 0.11) | 0.964 | **-0.17 (-0.26, -0.07)** | **<0.001** | -0.66 (-1.57, 0.25) | 0.296 | **0.15 (0.05, 0.25)** | **0.010** | **-0.18 (-0.26, -0.11)** | **<0.001** |
| -7 | 379 | 535 | **0.42 (0.19, 0.65)** | **<0.001** | 0.01 (-0.10, 0.12) | 0.964 | **-0.14 (-0.23, -0.06)** | **0.001** | -0.77 (-1.68, 0.14) | 0.239 | 0.09 (-0.00, 0.17) | 0.118 | **-0.16 (-0.23, -0.09)** | **<0.001** |
| -6 | 427 | 632 | **0.51 (0.28, 0.74)** | **<0.001** | -0.04 (-0.14, 0.06) | 0.762 | **-0.22 (-0.30, -0.14)** | **<0.001** | -0.80 (-1.60, 0.00) | 0.190 | **0.13 (0.05, 0.21)** | **0.008** | **-0.17 (-0.24, -0.11)** | **<0.001** |
| -5 | 486 | 691 | **0.49 (0.29, 0.70)** | **<0.001** | -0.05 (-0.15, 0.04) | 0.676 | **-0.22 (-0.29, -0.14)** | **<0.001** | **-0.88 (-1.62, -0.14)** | **0.019** | **0.14 (0.05, 0.22)** | **0.005** | **-0.20 (-0.27, -0.14)** | **<0.001** |
| -4 | 608 | 785 | **0.48 (0.28, 0.68)** | **<0.001** | -0.04 (-0.13, 0.05) | 0.724 | **-0.20 (-0.27, -0.13)** | **<0.001** | **-1.06 (-1.77, -0.36)** | **0.025** | **0.15 (0.08, 0.23)** | **<0.001** | **-0.19 (-0.25, -0.13)** | **<0.001** |
| -3 | 682 | 840 | **0.36 (0.18, 0.55)** | **<0.001** | **-0.13 (-0.21, -0.04)** | **0.014** | **-0.25 (-0.32, -0.18)** | **<0.001** | **-0.95 (-1.60, -0.30)** | **0.025** | **0.18 (0.11, 0.25)** | **<0.001** | **-0.25 (-0.30, -0.19)** | **<0.001** |
| -2 | 726 | 924 | **0.36 (0.17, 0.55)** | **<0.001** | **-0.15 (-0.23, -0.06)** | **<0.001** | **-0.25 (-0.32, -0.18)** | **<0.001** | **-1.06 (-1.65, -0.46)** | **0.019** | **0.17 (0.10, 0.24)** | **<0.001** | **-0.30 (-0.35, -0.24)** | **<0.001** |
| -1 | 661 | 871 | **0.41 (0.21, 0.62)** | **<0.001** | **-0.28 (-0.38, -0.20)** | **<0.001** | **-0.09 (-0.16, -0.02)** | **0.011** | -0.50 (-1.07, 0.11) | 0.239 | 0.06 (-0.02, 0.14) | 0.204 | **-0.25 (-0.31, -0.19)** | **<0.001** |
| 0 | 746 | 873 | **0.32 (0.11, 0.54)** | **<0.001** | **-0.30 (-0.40, -0.21)** | **<0.001** | **-0.20 (-0.27, -0.12)** | **<0.001** | -0.07 (-0.66, 0.51) | 0.802 | **0.08 (0.01, 0.16)** | **0.011** | **-0.24 (-0.30, -0.18)** | **<0.001** |
| Annual change | | | **0.11 (0.10, 0.13)** | **<0.001** | **-0.05 (-0.06, -0.04)** | **<0.001** | **-0.09 (-0.10, -0.09)** | **<0.001** | **-0.37 (-0.45, -0.30)** | **<0.001** | **0.04 (0.04, 0.05)** | **<0.001** | **-0.04 (-0.04, -0.03)** | **<0.001** |
| Difference in mean | | | **0.54 (0.39, 0.70)** | **<0.001** | **-0.38 (-0.46, -0.30)** | **<0.001** | **-0.25 (-0.31, -0.19)** | **<0.001** | -0.48 (-1.00, 0.04) | 0.069 | **0.16 (0.10, 0.22)** | **<0.001** | **-0.29 (-0.34, -0.25)** | **<0.001** |
| Difference in change | | | **0.02 (0.01, 0.04)** | **0.012** | **-0.09 (-0.10, -0.08)** | **<0.001** | **-0.01 (-0.02, -0.01)** | **<0.001** | **0.13 (0.02, 0.23)** | **0.017** | 0.01 (-0.00, 0.01) | 0.056 | **-0.02 (-0.02, -0.01)** | **<0.001** |

Difference was calculated as the mean of each measure in the death group minus that in the survivor group. Difference in change means the fixed effect of interaction between death and time. Models were adjusted for age at time 0 and sex.

**S Table 7.** Differences in psychological and social well-being between survivors and decedents in the 18 years preceding death: after PSM matchig.

|  | **Depression symptoms** | | **Loneliness** | | **Cognitive activity** | | **Social Activity** | | **Social Network** | | **Purpose of life** | |
| --- | --- | --- | --- | --- | --- | --- | --- | --- | --- | --- | --- | --- |
|  | Difference (95% CI) | *P* | Difference (95% CI) | *P* | Difference (95% CI) | *P* | Difference (95% CI) | *P* | Difference (95% CI) | *P* | Difference (95% CI) | *P* |
| Annual change | **0.12 (0.09, 0.14)** | **<0.001** | **0.04 (0.04, 0.05)** | **<0.001** | **-0.05 (-0.06, -0.04)** | **<0.001** | **-0.10 (-0.11, -0.09)** | **<0.001** | **-0.38 (-0.46, -0.30)** | **<0.001** | **-0.04 (-0.04, -0.03)** | **<0.001** |
| Difference in mean | **0.50 (0.33, 0.67)** | **<0.001** | **0.15 (0.09, 0.21)** | **<0.001** | **-0.32 (-0.40, -0.24)** | **<0.001** | **-0.22 (-0.28, -0.16)** | **<0.001** | -0.26 (-0.81, 0.29) | 0.349 | **-0.23 (-0.27, -0.18)** | **<0.001** |
| Difference in change | 0.01 (-0.00, 0.02) | 0.656 | 0.00 (-0.00, 0.01) | 0.703 | **-0.08 (-0.10, -0.07)** | **<0.001** | -0.01 (-0.02, 0.00) | 0.134 | **0.15 (0.01, 0.29)** | **0.033** | **-0.01 (-0.02, -0.01)** | **<0.001** |

Difference was calculated as the mean of each measure in the death group minus that in the survivor group. Difference in change means the fixed effect of interaction between death and time.

We performed 1:1 PSM matching with no-return sampling, and the matching variables were age at time0 and sex, and 750 pairs were eventually matched successfully and entered the analysis.

**S Table 8.** Differences in psychological and social well-being between survivors and decedents in the 18 years preceding death.

| **Year** | **No. of survivor** | **No. of death** | **Psychological well-being** | | **Social well-being** | |
| --- | --- | --- | --- | --- | --- | --- |
|  |  |  | Difference (95% CI) | *q-value* | Difference (95% CI) | *q-value* |
| -18 | 39 | 25 | -0.01 (-0.50, 0.47) | 0.953 | 0.00 (-0.36, 0.36) | 0.996 |
| -17 | 71 | 38 | -0.14 (-0.50, 0.21) | 0.484 | -0.24 (-0.55, 0.08) | 0.282 |
| -16 | 89 | 52 | -0.16 (-0.44, 0.11) | 0.359 | -0.08 (-0.32, 0.16) | 0.664 |
| -15 | 103 | 72 | -0.17 (-0.43, 0.09) | 0.359 | -0.15 (-0.36, 0.06) | 0.282 |
| -14 | 110 | 96 | -0.01 (-0.24, 0.21) | 0.953 | 0.00 (-0.21, 0.21) | 0.996 |
| -13 | 134 | 146 | -0.13 (-0.32, 0.06) | 0.359 | 0.02 (-0.15, 0.18) | 0.932 |
| -12 | 169 | 192 | -0.06 (-0.22, 0.11) | 0.496 | 0.03 (-0.11, 0.17) | 0.787 |
| -11 | 209 | 246 | -0.13 (-0.26, 0.01) | 0.203 | -0.04 (-0.17, 0.10) | 0.787 |
| -10 | 248 | 300 | -0.17 (-0.32, -0.02) | 0.148 | 0.02 (-0.10, 0.13) | 0.885 |
| -9 | 304 | 369 | **-0.24 (-0.37, -0.12)** | **0.019** | -0.13 (-0.24, -0.02) | 0.090 |
| -8 | 335 | 456 | **-0.29 (-0.41, -0.18)** | **0.019** | -0.11 (-0.22, -0.01) | 0.090 |
| -7 | 379 | 535 | **-0.24 (-0.34, -0.14)** | **0.019** | -0.10 (-0.21, -0.00) | 0.090 |
| -6 | 427 | 632 | **-0.30 (-0.40, -0.19)** | **0.019** | **-0.18 (-0.27, -0.09)** | **0.009** |
| -5 | 486 | 691 | **-0.30 (-0.40, -0.20)** | **0.019** | **-0.18 (-0.27, -0.09)** | **0.009** |
| -4 | 608 | 785 | **-0.31 (-0.40, -0.22)** | **0.019** | **-0.18 (-0.27, -0.10)** | **0.009** |
| -3 | 682 | 840 | **-0.32 (-0.40, -0.23)** | **0.019** | **-0.24 (-0.32, -0.16)** | **0.009** |
| -2 | 726 | 924 | **-0.35 (-0.43, -0.26)** | **0.019** | **-0.26 (-0.34, -0.18)** | **0.009** |
| -1 | 661 | 871 | **-0.30 (-0.39, -0.21)** | **0.019** | **-0.19 (-0.27, -0.11)** | **0.009** |
| 0 | 746 | 873 | **-0.31 (-0.40, -0.21)** | **0.019** | **-0.24 (-0.33, -0.16)** | **0.009** |
| Annual change | | | **-0.07 (-0.08, -0.06)** | **<0.001** | **-0.10 (-0.11, -0.09)** | **<0.001** |
| Difference in mean | | | **-0.41 (-0.48, -0.33)** | **<0.001** | **-0.31 (-0.37, -0.24)** | **<0.001** |
| Difference in change | | | **-0.02 (-0.03, -0.01)** | **<0.001** | **-0.03 (-0.04, -0.03)** | **<0.001** |

We standardized each score (z-scores) and created composite scores for psychological well-being (reverse-scored depressive symptoms + reverse-scored loneliness + purpose in life) and social well-being (social activity + social network + cognitive activity).

Difference was calculated as the mean of each measure in the death group minus that in the survivor group. Difference in change means the fixed effect of interaction between death and time. Models were adjusted for age at time 0 and sex.

**S Table 9.** Differences and 95% confidence intervals (CIs) in psychological and social well-being between survivors and decedents preceding death by sex.

| **Year** | **Depression symptoms** | | **Loneliness** | | **Social Activity** | |
| --- | --- | --- | --- | --- | --- | --- |
|  | Female | Male | Female | Male | Female | Male |
| -18 | -0.40 (-1.70, 0.90) | -0.42 (-1.29, 0.44) | 0.08 (-0.41, 0.57) | -0.17 (-1.23, 0.89) | -0.16 (-0.53, 0.21) | 0.19 (-1.33, 1.71) |
| -17 | -0.07 (-0.80, 0.65) | 0.70 (-0.93, 2.28) | 0.04 (-0.29, 0.38) | -0.27 (-1.00, 0.47) | -0.27 (-0.55, 0.01) | -0.08 (-0.72, 0.56) |
| -16 | 0.56 (-0.07, 1.19) | 0.01 (-0.93, 0.95) | 0.12 (-0.18, 0.42) | -0.38 (-0.89, 0.14) | -0.03 (-0.26, 0.19) | -0.05 (-0.38, 0.48) |
| -15 | 0.19 (-0.40, 0.78) | 0.55 (-0.40, 1.50) | 0.22 (-0.05, 0.49) | 0.27 (-0.36, 0.91) | -0.16 (-0.36, 0.05) | -0.25 (-0.71, 0.21) |
| -14 | -0.03 (-0.56, 0.49) | 0.21 (-0.53, 0.95) | 0.06 (-0.18, 0.31) | -0.04 (-0.48, 0.40) | -0.10 (-0.29, 0.08) | 0.06 (-0.31, 0.43) |
| -13 | 0.36 (-0.11, 0.82) | 0.45 (-0.25, 1.15) | 0.07 (-0.12, 0.26) | -0.12 (-0.50, 0.26) | -0.06 (-0.21, 0.10) | -0.21 (-0.54, 0.13) |
| -12 | 0.04 (-0.36, 0.45) | 0.03 (-0.37, 0.44) | -0.02 (-0.20, 0.15) | 0.06 (-0.23, 0.36) | -0.06 (-0.20, 0.08) | -0.13 (-0.42, 0.16) |
| -11 | 0.36 (-0.03, 0.74) | 0.29 (-0.24, 0.82) | 0.08 (-0.06, 0.23) | -0.12 (-0.39, 0.15) | -0.13 (-0.26, 0.01) | -0.18 (-0.47, 0.12) |
| -10 | 0.15 (-0.17, 0.47) | 0.48 (0.02, 0.94) | 0.07 (-0.06, 0.21) | 0.07 (-0.20, 0.33) | -0.05 (-0.17, 0.07) | -0.20 (-0.41, 0.01) |
| -9 | **0.37 (0.08, 0.67)*** | 0.28 (-0.16, 0.72) | **0.12 (0.01, 0.24)*** | 0.06 (-0.19, 0.31) | **-0.16 (-0.27, -0.05)*** | -0.18 (-0.40, 0.04) |
| -8 | **0.62 (0.32, 0.91)*** | 0.22 (-0.16, 0.60) | **0.16 (0.05, 0.28)*** | 0.10 (-0.11, 0.33) | **-0.14 (-0.25, -0.04)*** | **-0.24 (-0.42, -0.07)*** |
| -7 | **0.45 (0.18, 0.73)*** | 0.33 (-0.07, 0.73) | **0.10 (0.00, 0.20)*** | 0.03 (-0.15, 0.21) | **-0.13 (-0.23, -0.04)*** | **-0.20 (-0.39, -0.01)*** |
| -6 | **0.50 (0.24, 0.76)*** | **0.57 (0.18, 1.03)*** | **0.14 (0.04, 0.23)*** | 0.10 (-0.07, 0.28) | **-0.20 (-0.29, -0.11)*** | **-0.30 (-0.47, -0.12)*** |
| -5 | **0.51 (0.27, 0.76)*** | **0.43 (0.05, 0.81)*** | **0.15 (0.05, 0.24)*** | 0.10 (-0.07, 0.27) | **-0.22 (-0.30, -0.13)*** | **-0.22 (-0.38, -0.06)*** |
| -4 | **0.51 (0.27, 0.75)*** | **0.39 (0.06, 0.73)*** | **0.17 (0.08, 0.26)*** | 0.20 (-0.05, 0.26) | **-0.18 (-0.26, -0.10)*** | **-0.27 (-0.42, -0.13)*** |
| -3 | **0.43 (0.20, 0.66)*** | 0.14 (-0.14, 0.42) | **0.17 (0.08, 0.25)*** | **0.21 (0.08, 0.35)*** | **-0.24 (-0.32, -0.16)*** | **-0.30 (-0.44, -0.16)*** |
| -2 | **0.37 (0.14, 0.60)*** | **0.35 (0.04, 0.66)*** | **0.15 (0.07, 0.23)*** | **0.23 (0.10, 0.37)*** | **-0.26 (-0.34, -0.18)*** | **-0.21 (-0.35, -0.07)*** |
| -1 | **0.32 (0.08, 0.56)*** | **0.68 (0.32, 1.04)*** | **0.04 (-0.05, 0.12)*** | **0.17 (0.04, 0.31)*** | -0.04 (-0.12, 0.04) | **-0.20 (-0.34, -0.07)*** |
| 0 | **0.36 (0.11, 0.61)*** | **0.48 (0.10, 0.86)*** | **0.06 (-0.02, 0.15)*** | **0.21 (0.07, 0.35)*** | **-0.19 (-0.27, -0.11)*** | **-0.18 (-0.31, -0.05)*** |
| Annual change | **0.11 (0.08, 0.13)*** | **0.14 (0.11, 0.18)*** | **0.05 (0.04, 0.05)*** | **0.03 (0.02, 0.04)*** | **-0.09 (-0.10, -0.09)*** | **-0.09 (-0.10, -0.08)*** |
| Difference in mean | **0.54 (0.36, 0.71)*** | **0.63 (0.35, 0.91)*** | **0.14 (0.08, 0.21)*** | **0.24 (0.13, 0.35)*** | **-0.23 (-0.29, -0.16)*** | **-0.27 (-0.38, -0.16)*** |
| Difference in change | 0.01 (-0.01, 0.03) | **0.04 (0.01, 0.07)*** | 0.00 (-0.00, 0.01) | **0.02 (0.01, 0.03)*** | **-0.01 (-0.02, -0.01)*** | **-0.02 (-0.03, -0.01)*** |
| P for interaction | 0.061 | | 0.098 | | 0.259 | |

Difference was calculated as the mean of each measure in the death group minus that in the survivor group. Difference in change means the fixed effect of interaction between death and time. Models were adjusted for age at time 0.

**S Table 10.** Differences and 95% confidence intervals (CIs) in psychological and social well-being between survivors and decedents preceding death by sex.

| **Year** | **Social Network** | | **Cognitive activity** | | **Purpose of life** | |
| --- | --- | --- | --- | --- | --- | --- |
|  | Female | Male | Female | Male | Female | Male |
| -18 | 1.88 (-1.05, 4.80) | -7.93 (-17.33,1.45) | -0.06 (-0.56, 0.44) | 0.33 (-1.35, 2.02) | -0.34 (-0.70, 0.03) | 0.07 (-0.87, 1.00) |
| -17 | -0.33 (-4.36, 3.69) | -4.48 (-9.43, 0.44) | -0.03 (-0.33, 0.27) | 0.01 (-0.75, 0.78) | -0.20 (-0.43, 0.04) | -0.29 (-1.04, 0.46) |
| -16 | -1.53 (-4.21, 1.16) | -2.25 (-6.52, 1.99) | 0.04 (-0.23, 0.31) | 0.15 (-0.41, 0.71) | -0.14 (-0.35, 0.07) | 0.03 (-0.35, 0.41) |
| -15 | -0.79 (-3.17, 1.58) | -2.65 (-6.72, 1.41) | -0.01 (-0.26, 0.23) | -0.11 (-0.56, 0.34) | -0.05 (-0.23, 0.13) | -0.11 (-0.51, 0.29) |
| -14 | -0.66 (-2.76, 1.45) | -0.14 (-6.08, 5.80) | 0.15 (-0.07, 0.36) | -0.03 (-0.44, 0.38) | -0.02 (-0.18, 0.14) | -0.11 (-0.45, 0.23) |
| -13 | 0.10 (-1.57, 1.76) | 1.67 (-2.99, 6.35) | 0.02 (-0.17, 0.20) | 0.09 (-0.24, 0.43) | -0.15 (-0.29, -0.01) | -0.15 (-0.47, 0.16) |
| -12 | 1.54 (-0.07, 3.01) | -0.40 (-3.61, 2.82) | 0.02 (-0.16, 0.19) | 0.16 (-0.09, 0.41) | -0.10 (-0.22, 0.02) | -0.23 (-0.45, 0.01) |
| -11 | 0.64 (-0. 73, 2.00) | -1.36 (-4.83, 2.11) | 0.08 (-0.07, 0.24) | -0.01 (-0.27, 0.26) | **-0.16 (-0.26, -0.04)*** | -0.19 (-0.41, 0.04) |
| -10 | 0.32 (-0.90, 1.55) | 0.35 (-2.27, 2.97) | 0.10 (-0.05, 0.25) | -0.01 (-0.23, 0.21) | **-0.09 (-0.19, -0.01)*** | -0.02 (-0.22, 0.18) |
| -9 | -0.88 (-1.95, 0.18) | -0.44 (-2.49, 1.61) | -0.02 (-0.15, 0.12) | 0.03 (-0.19, 0.26) | **-0.16 (-0.25, -0.07)*** | -0.14 (-0.33, 0.05) |
| -8 | -0.27 (-1.30, 0.77) | **-2.01 (-3.91, -0.11)*** | -0.02 (-0.14, 0.12) | 0.01 (-0.21, 0.21) | **-0.17 (-0.25, -0.08)*** | **-0.23 (-0.39, -0.07)*** |
| -7 | -0.42 (-1.47, 0.62) | **-2.10 (-3.95, -0.25)*** | 0.02 (-0.10, 0.14) | -0.05 (-0.26, 0.16) | **-0.15 (-0.23, -0.07)*** | **-0.22 (-0.38, -0.06)*** |
| -6 | -0.60 (-1.50, 0.30) | **-1.66 (-3.39, -0.07)*** | -0.04 (-0.15, 0.08) | -0.07 (-0.26, 0.12) | **-0.17 (-0.24, -0.09)*** | **-0.20 (-0.34, -0.05)*** |
| -5 | -0.68 (-1.53, 0.17) | **-1.55 (-3.03, -0.08)*** | -0.05 (-0.16, 0.06) | -0.08 (-0.24, 0.08) | **-0.19 (-0.26, -0.11)*** | **-0.26 (-0.39, -0.13)*** |
| -4 | **-1.09 (-1.84, -0.33)*** | -1.01 (-2.70, 0.68) | -0.04 (-0.15, 0.06) | -0.05 (-0.21, 0.11) | **-0.20 (-0.27, -0.13)*** | **-0.16 (-0.28, -0.05)*** |
| -3 | **-1.00 (-1.75, -0.25)*** | -0.83 (-2.14, 0.47) | **-0.13 (-0.23, -0.03)*** | -0.121 (-0.27, 0.05) | **-0.24 (-0.30, -0.18)*** | **-0.27 (-0.38, -0.15)*** |
| -2 | **-1.02 (-1.72, -0.32)*** | -1.15 (-2.29, 0.01) | **-0.15 (-0.25, -0.05)*** | -0.14 (-0.29, 0.01) | **-0.29 (-0.35, -0.22)*** | **-0.32 (-0.42, -0.21)*** |
| -1 | -0.50 (-1.15, 0.15) | 0.12 (-0.97, 1.22) | **-0.27 (-0.37, -0.16)*** | **-0.25 (-0.41, -0.08)*** | **-0.23 (-0.30, -0.16)*** | **-0.33 (-0.43, -0.22)*** |
| 0 | 0.16 (-0.47, 0.79) | -0.30 (-1.43, 0.83) | **-0.31 (-0.42, -0.21)*** | **-0.31 (-0.48, -0.15)*** | **-0.22 (-0.29, -0.16)*** | **-0.33 (-0.43, -0.22)*** |
| Annual change | **-0.34 (-0.42, -0.25)*** | **-0.50 (-0.67, -0.34)*** | **-0.05 (-0.06, -0.04)*** | **-0.05 (-0.06, -0.03)*** | **-0.04 (-0.04, -0.03)*** | **-0.04 (-0.05, -0.03)*** |
| Difference in mean | -0.39 (-0.96, 0.18) | -0.22 (-1.18, 0.75) | **-0.38 (-0.47, -0.29)*** | **-0.36 (-0.50, -0.21)*** | **-0.28 (-0.33, -0.23)*** | **-0.35 (-0.44, -0.26)*** |
| Difference in change | **0.01 (0.00, 0.02)*** | **0.03 (0.01, 0.05)*** | **-0.09 (-0.10, -0.08)*** | **-0.10 (-0.13, -0.07)*** | **-0.01 (-0.02, -0.01)*** | **-0.02 (-0.03, -0.01)*** |
| P for interaction | 0.317 | | 0.474 | | **0.045** | |

Difference was calculated as the mean of each measure in the death group minus that in the survivor group. Difference in change means the fixed effect of interaction between death and time. Models were adjusted for age at time 0.

**S Table 11.** Differences in psychological and social well-being change at the pre-terminal and terminal time among decedents (N=1,119).

| **Year** | **Depression symptoms** | **Loneliness** | **Cognitive activity** | **Social Activity** | **Social Network** | **Purpose of life** |
| --- | --- | --- | --- | --- | --- | --- |
|  | β (95% CI) | β (95% CI) | β (95% CI) | β (95% CI) | β (95% CI) | β (95% CI) |
| Change point (year) | -9 | -6 | -10 | -9 | -4 | -11 |
| Annual change at pre-terminal time | 0.01 (-0.02, 0.04) | 0.01 (0.00, 0.02)* | -0.03 (-0.05, -0.01)* | -0.04 (-0.05, -0.03)* | -0.14 (-0.20, -0.07)* | -0.02 (-0.04, -0.01)* |
| Annual change at terminal time | 0.08 (0.07, 0.10)* | 0.04 (0.03, 0.04)* | -0.09 (-0.10, -0.08)* | -0.08 (-0.09, -0.08)* | -0.22 (-0.31, -0.13)* | -0.04 (-0.04, -0.03)* |
| Difference in slope before and after change point | 0.08 (0.04, 0.13)* | 0.02 (0.01, 0.03)* | -0.06 (-0.09, -0.04)* | -0.05 (-0.06, -0.04)* | -0.06 (-0.16, 0.05) | -0.02 (-0.03, -0.01)* |

Models were adjusted for age at time 0 and sex.

***** *P*< 0.05.


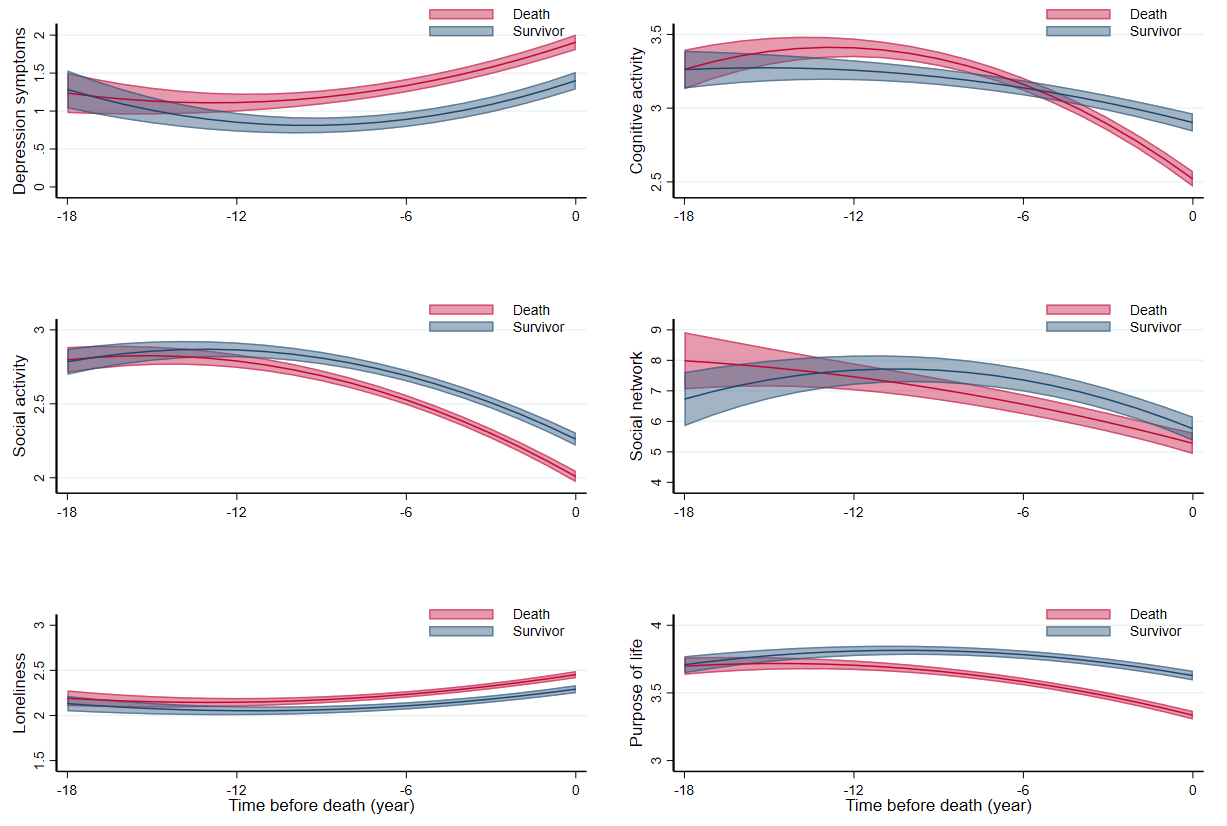


**S Figure 2.** Psychological and social well-being trajectory by mortality status (with ≥3 repeated measurements).

Model adjusted for age at time 0 and sex.


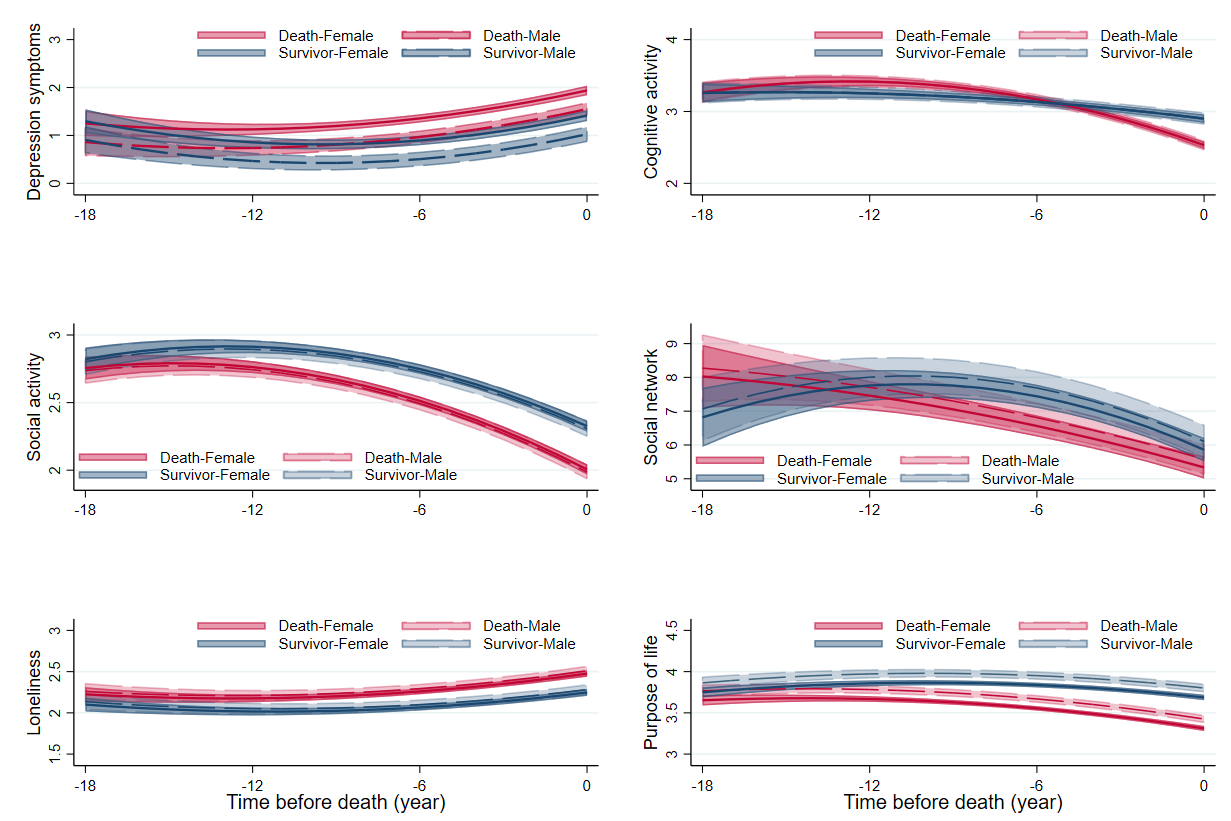
**S Figure 3.** Psychological and social well-being trajectory preceding death stratified by sex.


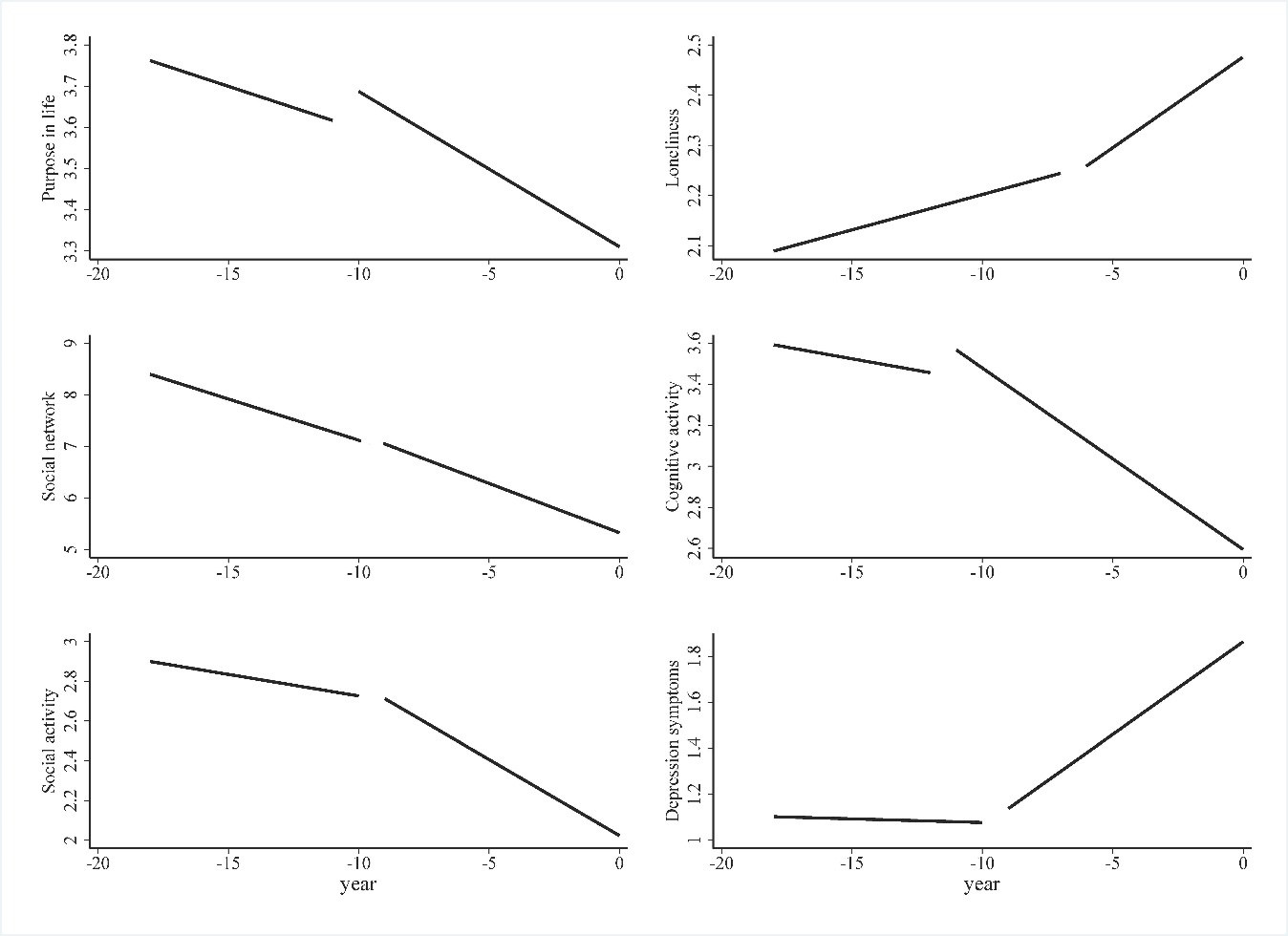
**S Figure 4.** Psychological and social well-being trajectory preceding death among decedents: results from the change-point mixed-effects model.
